# Spatial environmental complexity mediates sexual conflict and sexual selection in *Drosophila melanogaster*

**DOI:** 10.1002/ece3.4932

**Published:** 2019-01-30

**Authors:** Heather L. Malek, Tristan A. F. Long

**Affiliations:** ^1^ Department of Biology Wilfrid Laurier University Waterloo Ontario Canada

**Keywords:** environmental complexity, mate choice, mating systems, sexual conflict, sexual selection, spatial complexity

## Abstract

Sexual selection is an important agent of evolutionary change, but the strength and direction of selection often vary over space and time. One potential source of heterogeneity may lie in the opportunity for male–male and/or male–female interactions imposed by the spatial environment. It has been suggested that increased spatial complexity permits sexual selection to act in a complementary fashion with natural selection (hastening the loss of deleterious alleles and/or promoting the spread of beneficial alleles) via two (not mutually exclusive) pathways. In the first scenario, sexual selection potentially acts more strongly on males in complex environments, allowing males of greater genetic “quality” a greater chance of outcompeting rivals, with benefits manifested indirectly in offspring. In the second scenario, increased spatial complexity reduces opportunities for males to antagonistically harm females, allowing females (especially those of greater potential fecundities) to achieve greater reproductive success (direct fitness benefits). Here, using *Drosophila melanogaster*, we explore the importance of these mechanisms by measuring direct and indirect fitness of females housed in simple vial environments or in vials in which spatial complexity has been increased. We find strong evidence in favor of the female conflict‐mediated pathway as individuals in complex environments remated less frequently and produced more offspring than those housed in a simpler spatial environment, but no difference in the fitness of sons or daughters. We discuss these results in the context of other recent studies and what they mean for our understanding of how sexual selection operates.

## 1 INTRODUCTION

Patterns of nonrandom mating that arise as a consequence of either intra‐ or intersexual selection can have potentially dramatic consequences for a species’ evolutionary trajectory. Understanding the factors that shape the outcome of these two (not necessarily independent) forms of sexual selection (and, by extension, their often‐complicated relationship with respect to natural selection) is of great importance to evolutionary biologists (Chenoweth, Appleton, Allen, & Rundle, 2015; Hollis, Fierst, & Houle, 2009). In both intra‐ and intersexual selection, one potentially important mediating factor is the physical characteristics of the environment in which organisms are located. In this study, we set out to examine how changes in spatial complexity potentially alter the outcome of mating dynamics in *Drosophila melanogaster*, a model species for the study of sexual selection and conflict.

It has long been recognized that the degree of complexity in an environment can influence the speed and/or direction of adaptive evolution via natural selection (Łukasik, Radwan, & Tomkins, 2006). Environmental heterogeneity can generate different and more varied selective pressures than those arising in a simpler, homogenous environment (Miller & Svensson, 2014). Adaptations are, by definition “phenotypic variants that result in the highest fitness among a specific set of variants in a given environment” (Reeve & Sherman, 1993). It follows that if there is environmental uniformity, directional selection will act more efficiently on traits, than when there is spatial and/or temporal heterogeneity resulting in distinct selective regimes (Long, Rowe, & Agrawal, 2013). It is also becoming increasingly evident that the operation of sexual selection may also be strongly shaped by the complexity of the local environment, by altering the dynamics of male–male and/or male–female interactions. The spatial ecology of an environment can potentially affect the frequency and types of intraspecific interactions, which can have important consequences for how sexual selection and sexual conflict operate.

Environmental spatial complexity may involve the presence of physical barriers or obstacles, which limit the frequency of encounters between both potential mates and/or rivals. This change in encounter rates can directly shape the type of mating strategies adopted by males. To understand the potential importance of spatial complexity, let us first consider another factor that can influence male–male encounter rates: population density (reviewed in Kokko & Rankin, 2006). The density of a population can influence mating decisions made by males and their investment strategies at the pre‐ and postcopulatory levels. Selection may be strengthened at high densities if males engage in “scramble” competitions (Parker, 2000; Thornhill & Alcock, 1983) and access to females depends largely on an individual's ability to exclude rivals (Arak, 1983; Kokko & Rankin, 2006). Alternatively, selection on males may be greater at low densities, if only a few “superior” individuals are capable of locating and successfully mating with multiple, widely dispersed females (Sharp & Agrawal, 2008). If a major factor influencing male behavior is encounter rate, then spatially complex environments may be functionally equivalent to that of a low‐density population. In a short‐term assay conducted using *D. melanogaster* males placed in two vastly different sized environments, MacLellan, Whitlock, and Rundle (2009) found that the strength of sexual selection acting against males possessing visible mutations (with potentially deleterious effects) was greater in larger chambers, presumably due to the increased search effort required to find females. As this assay ran for only 24 hr and most females mated only once, most of this variation was attributed to searching efficiency and precopulatory traits, rather than traits involved in pre‐ or postcopulatory male–male contest competition (or female choice, for that matter). Variation in male–male encounter rates may also shape traits involved in postcopulatory sexual selection. For instance, in high‐density environments where encounter rates and polyandry are high, males experience a greater risk of sperm competition (Jarrige, Riemann, Goubault, & Schmoll, 2015; Long & Montgomerie, 2006). Bretman, Fricke, and Chapman (2009) found that male *D. melanogaster* housed with rivals prior to encountering females mated for longer and sired a greater fraction of offspring in twice‐mated females. However, increased investment into sperm and ejaculates is costly and may only be beneficial in crowded environments where there is heightened postcopulatory competition (Parker & Pizzari, 2010). Thus, there is great potential for variation in spatial ecology to influence the strength of *intra*sexual selection operating in a population.

The spatial complexity of an environment can also impact *inter*sexual selection. The decision whether or not to mate (and with whom) can be influenced by numerous factors including individual condition (which might be associated with age and/or nutritional levels), effectiveness in mate assessment and sampling strategies, access to mates, the intensity of male–male competition, the presence of rival females, and of predation risk, all of which can be potentially vary with ecological context (Kokko & Rankin, 2006; Miller & Svensson, 2014). In environments with higher spatial complexity, if encounter rates between males and females are reduced, both sexes may adjust their behavioral strategies to account for increased search costs (Hack, 1998; Parker, 1983). Search costs often necessitate a trade‐off against mate choosiness, as more energy is expended in finding a potential mate, and there is a greater cost of rejecting that individual, potentially leading to the weakening of sexual selection in a low‐density/spatially complex environments (Barry & Kokko, 2010; Booksmythe, Jennions, & Backwell, 2010; Lindström & Lehtonen, 2013). An increase in sampling costs can contribute to individual variation in female mate choice, resulting in changes in the strength and/or direction of sexual selection. Such heterogeneity in sexual selection can influence the amount of standing genetic variation present in a population, compared to what would be observed if directional selection was allowed to proceed in an unimpeded fashion (Falconer & Mackay, 1996; Jennions & Petrie, 1997; Widemo & Sæther, 1999). However, it is also possible that the strength of intersexual selection exerted by females may become stronger in more spatially complex environments as there are fewer opportunities for rival males to engage in competitive interactions that might otherwise interfere with female mate choice (Wong & Candolin, 2005). Thus, environmental spatial complexity has great potential to mediate the shape of mating systems and to direct the evolutionary trajectory that a species follows.

The expression of intersexual conflict in a population may also be shaped by its environment. As a result of different (and often incompatible) fitness‐maximizing strategies over mating rates, males in many species have evolved numerous behavioral and morphological traits that benefit their own fitness by manipulating females (Arnqvist & Rowe, 2005; Clutton‐Brock & Parker, 1995). However, through their selfish actions, these males cause females direct harm or influence them to behave suboptimally, ultimately reducing their lifetime reproductive success. For instance, in water striders, *Gerris odontogaster*, males and females often engage in an aggressive precopulatory struggle, with females attempting to dislodge males who are attempting to copulate with them (Arnqvist, 1989). This resistance is costly to females, and, as the local density of males increases, females adjust their behaviors to exhibit less reluctance to mate to avoid increased harassment‐associated costs (Arnqvist, 1992). In the seaweed fly, *Coelopa frigida,* the intensity of sexual conflict (measured as the frequency of male harassment of females) depends on the presence (and type) of algae present in their environment (Edward & Gilburn, 2007). In *D. melanogaster*, males harm females directly through harassment during courtship (Partridge & Fowler, 1990), physical damage during copulation (Kamimura, 2007), and/or the activity of products transferred in the male's seminal fluid (Fowler & Partridge, 1989; Wolfner, 2009). This effect is exacerbated when the males that are the most successful at courting and mating also induce the greatest harm to their mates (Friberg & Arnqvist, 2003; Pitnick & García‐González, 2002). Furthermore, when harmful male attention is directed toward those females within a population with the greatest potential fecundity, this can interfere with the process of adaptive evolution (Chenoweth et al., 2015; Long, Pischedda, Stewart, & Rice, 2009). The strength of conflict is often expected to be greater at high population densities (Martin & Hosken, 2003), and it is hypothesized that increased environmental spatial complexity may help females avoid conflict‐associated costs by forcing males to spend more time locating a mate, providing potential “refuges” for females from persistent male courtship and harassment (Byrne, Rice, & Rice, 2008; Yun, Chen, Singh, Agrawal, & Rundle, 2017). A reduction in male harassment may allow females to invest more energy into feeding and being “choosier” during mate selection (Heubel & Plath, 2008; Köhler et al., 2011), but might alternatively result in females exhibiting less resistance (and thus greater remating) in association with the lower male encounter rate (Kokko & Rankin, 2006). The consequences of mating with coercive males may also be reflected in offspring fitness when males pass on alleles with sexually antagonistic effects, benefitting sons while reducing daughter fitness (Berger et al., 2016; Chippindale, Gibson, & Rice, 2001) with the long‐term co‐evolutionary consequences depending on the strength of the pleiotropic relationship interaction between the inter‐ and intralocus sexual conflict (Pennell, Haas, Morrow, & Doorn, 2016).

The scenarios described above are strongly suggestive that environmental complexity potentially mediates mating‐related behaviors and the operation of sexual selection, and at the same time infers two different (but not mutually exclusive) pathways in which it may influence adaptive evolution. In the first pathway, increased environmental complexity may result in changes in how inter‐ and/or intrasexual selection acts on males. Those carrying deleterious mutations have an increased selective disadvantage in more complex environment (as in MacLellan et al., 2009), thereby enhancing the efficiency with which sexual selection purges them from the population's gene pool (Hollis et al., 2009; Whitlock & Agrawal, 2009). In the second pathway, increased spatial complexity restricts the ability of males to aggressively court and injure females in the population. Free from (some) of this “cost of attractiveness” (sensu Long et al., 2009), these individuals are able to make a larger contribution of offspring to the next generation, potentially resulting in an increased rate that beneficial alleles spread through the population (Chenoweth et al., 2015). Both these mechanisms potentially result in increased population fitness, either through the production of higher quality offspring or through increased mean female fecundity. To better understand how environmental complexity mediates sexual selection and its consequences, it is necessary to conduct experiments in which both possible pathways are examined. Here, we set out to conduct such empirical studies by exploring male–female interactions in *D. melanogaster* with specific attention paid to their role in mediating male‐induced harm and remating rates, and how these both potentially contribute to the fitness of offspring in the next generation.

## 2 MATERIALS AND METHODS

### 2.1 Population history and maintenance

The source of focal *D. melanogaster* flies used in our assays is the *Ives* (hereafter “IV”), population, a large, outbred wild‐type stock that originated from a sample of 200 mated females caught in Amherst MA, USA in 1975 (Rose, 1984). Our assay also used “competitor” flies from both the IV‐*bwD* and IV‐*bw* populations, which were created by introgressing (via repeated backcrossing) the dominant brown‐eyed allele, *bwD* and the recessive brown‐eyed allele, *bw^1^* (respectively) into the IV genetic background.

These populations are maintained at large size (~3,500 adults/generation) on a 14‐day discrete nonoverlapping culture cycle, where flies are cultured *en masse* (under light CO_2_ anesthesia) in vials containing 10 ml of a banana killed‐yeast agar media (Rose, 1984). All populations are kept at a density of ~100 eggs/vial and are housed at 25ºC, 60% RH and exposed to a 12‐hr‐L:12‐hr‐D diurnal light cycle (Martin & Long, 2015).

### 2.2 Measuring female remating rates and offspring production in environments of different complexity

**Figure 1:**
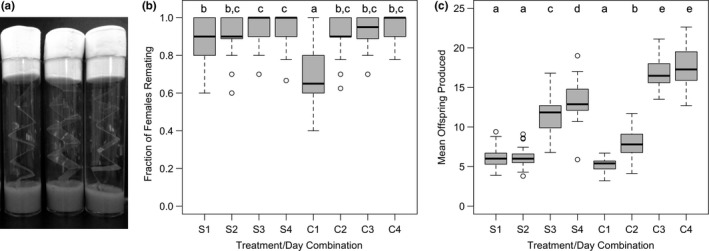
(a) Standard *Drosophila* culture vials used in this assay each containing 10 male and female 10 flies, agar/banana/killed‐yeast media and the accordion‐style acetate inserts used in our experimental treatments to increase environmental spatial complexity. (b and c) Boxplots illustrating (b) the fraction of female *Drosophila melanogaster* in a vial that remated and (c) their mean offspring production in either simple (S) or environmentally complex (C) chambers,measured on each of the 4 days of the assay. The boxes enclose the middle 50% of data (interquartile range, IQR), with the location of the median represented by a horizontal line. Values >±1.5 × the IQR outside the box are considered outliers and depicted as open circles. Whiskers extend to the largest and smallest values that are not outliers. Boxplots that are not sharing a letter have significantly different means

In this assay, we set out to determine whether housing females in environments differing in their spatial complexity influenced remating rates and/or female fecundity. All flies used in this assay were collected as virgins (within 8 hr of eclosion) and housed in same‐sex groups of 10 for 3–4 days prior to the experiment. The experiment began by combining 240 sets of 10 female IV flies (without anesthesia) with an equal number of IV‐*bwD* males for a period of 3 hr, which allows sufficient time for all females to mate once (TAFL, pers. obs.). Next, using light anesthesia, the IV‐*bwD* males were removed and replaced with sets of 10 IV males. Into half of these vials (“experimental treatment”), we also added a clear strip of acetate (~1.5 × 13 cm) folded “accordion‐style” in order to increase the surface area within the vial (Figure 1a). In the remaining 120 vials (control treatment), no acetate strip was added. On each of the subsequent 4 days, we haphazardly removed females from 30 of the vials in each treatment and transferred them (under light anesthesia) into individual test tubes containing ~3 ml of fresh media, the surface of which has been cut to promote oviposition (Rice et al., 2005). Females were left to oviposit for 23 hr before being discarded. The test tubes were incubated under standard conditions for 14 days, at which time the number, and eye color, of all offspring in each tube was tallied. Test tubes that contained wild‐type offspring indicate that the female had remated.

### 2.3 Assay to examine potential impact of environmental complexity on offspring fitness

In these subsequent assays, we set out to determine whether the increased environmental spatial complexity altered the outcome of mate choice, resulting in higher offspring fitness, compared to those females in the control vials. For this experiment, we established, using the same protocol described above, a new set of 240 vials each consisting of 10 IV females (that had just been mated to IV‐*bwD* males) and 10 IV males. Half of the vials contained a folded strip of acetate, while the other half did not. On each of the next four days, we haphazardly selected 30 vials from each treatment and transferred (anesthetised) females into a small egg‐laying chamber overnight. The following morning, up to 50 eggs were collected from each of these chambers and transferred to vials containing fresh media. We added a sufficient number of similarly aged IV‐*bw* eggs so that each vial contained 100 eggs (thereby matching typical culture conditions). This experimental setup was replicated twice, to independently measure both daughter and son fitnesses, using the protocols described below.

#### 2.3.1 Fitness assay of daughters

For each of the four temporally offset sets of vials, we collected, 14 days after their creation, five wild‐type females from each vial and transferred them to individual test tubes for ~18 hr to oviposit before being discarded. Test tubes were returned to the incubator for an additional 14 days at which point all eclosed adult flies were removed and counted. This count represents the fitness of daughters produced as a result of their mothers mating with an IV male.

#### 2.3.2 Fitness assay of sons

Starting ~9 days after their setup, we haphazardly collected a single, virgin, wild‐type IV male (within 8 hr of their eclosion) from each vial in our four temporally offset sets of vials. This male was individually placed into a new vial containing 9 similarly aged IV‐*bw* males and 10 IV‐*bw* virgin females. Flies were left in these vials for a period of 48 hr at which time the IV‐*bw* females were anesthetized and transferred into individual test tubes for an additional 24 hr before being discarded. Test tubes were incubated for 14 days and the number and phenotype of eclosed adults were tallied in order to determine both the total number of offspring sired by the wild‐type male and the total the number of females that the target male had mated with; these represent meaningful indices of the fitness of sons produced as a result of their mothers mating with an IV male.

### 2.4 Statistical analysis

All statistical analyses were performed using R.3.3.2 (R Core Team). In all analyses, vial represents the unit of replication, and in all models (unless specified otherwise), spatial environment treatment, assay day, and their interaction were included as independent variables. The magnitude of differences between groups from different spatial environments on specific days was quantified using Cohen's d or Cliff's delta effect size statistics using functions in the *effsize* package (Torchiano, 2017). To analyze the effects of environmental complexity on adult female remating rates over the 4 days of the experiment, we constructed generalized linear models (GLMs), with a quasibinomial error distributions, where our response variable was the number of females in a vial that produced offspring whose eye phenotype was *wild‐type *(which indicated remating had occurred), where spatial treatment, day, and their interaction were the independent variables. To analyze whether variation in offspring production was associated with the spatial environment treatment and/or day, we calculated the mean number of offspring produced by females in each vial and used that as the response variable in a GLM with Gaussian error distributions where spatial treatment, day, and their interaction were the independent variables. The significance of independent variables in our GLMs was determined using the *ANOVA* function in the *car* package (Fox & Weisberg, 2011). The location of specific differences between groups was assessed, where necessary, using a Tukey HSD post hoc test using the *glht* function in the *multcomp* package (Hothorn, Bretz, & Westfall, 2008). We also examined whether there were any differences in sperm displacement/use across treatments/days by computing the mean P2 values (the fraction of offspring sired by the wild‐type male(s) averaged across each vial), for all females, as well as for only those females that were deemed to have remated (by the presence of *wild‐type* offspring in their offspring). As these vial–mean ratio data had non‐normal distributions, we employed the Scheirer–Ray–Hare extension of the Kruskal–Wallis test (Sokal & Rohlf, 1995) on a two‐factor ANOVA to examine the effects of environmental complexity, assay day, and their interaction. Effect sizes for comparisons between treatments for each day were determined using Cliff's delta method.

In our analysis of daughter fitness data, we constructed a GLM with Gaussian error distributions where the response variable was the average offspring produced by the five females per vial that were sampled.

For sons, we quantified fitness in two different ways: first as their success at mating with females (measured as the fraction of females in a vial that produced wild‐type offspring) and secondly as the total number of grandchildren sired (measured as the sum of all wild‐type offspring produced by females from the same vial). We analyzed both the “absolute” values for these metrics, as well as their “relative” values (by diving all values by the greatest absolute fitness value observed). For absolute fitness metrics, we constructed GLMs (with quasibinomial, and quasipoisson error distributions, respectively) with spatial environment type, assay day, and their interaction as independent variables, with their statistical significance determined using the methods described above. For relative fitness metrics, we analyzed the effects of spatial environment type, assay day, and their interaction on these ratio variables using the nonparametric Scheirer–Ray–Hare method.

## 3 RESULTS

### 3.1 Remating rates and female offspring production

**Figure 2:**
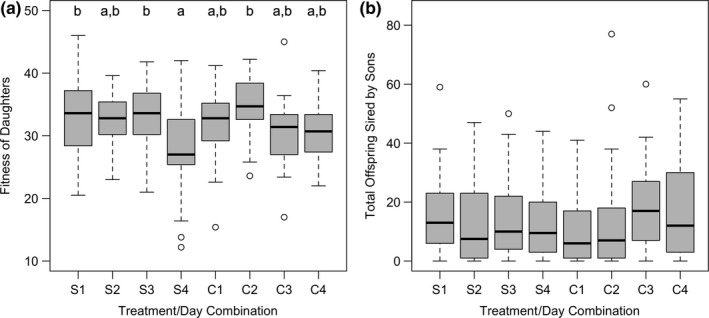
(a) Boxplots illustrating the offspring production of *Drosophila melanogaster* daughters from a parent generation housed for 1–4 days in female in simple (S) or environmentally complex (C) chambers measured on each of the 4 days of the assay. Boxplots that are not sharing a letter have significantly different means. (b) Boxplots illustrating the offspring production of sons from a parent generation housed for 1–4 days in simple (S) or environmentally complex (C) chambers measured on each of the 4 days of the assay. Boxplot components are as described in Figure 1

In our first assay we measured, over the course of 4 days, the potential impact of differences in environmental spatial complexity by evaluating remating rates of females and their fecundity. We observed a significant interaction between remating rates of females and the day of the assay (Table 1a), with females in the simpler environment exhibiting greater rates of remating in the first 24 hr of the assay (Table 2, Figure 1b). This difference between treatments was not detected for females who were measured 48, 72, or 96 hr after the start of the assay (Figure 2). When we analyzed the number of offspring produced by females housed in either a simple or complex environment, we saw a significant effect of treatment, of day and their interaction (Table 1b). In both environments females produced, on average, more offspring on later days, but, starting on Day 2 of the assay, female offspring production was higher in the complex environment offspring than in the simpler environment (Figure 1c, Table 3).

**Table 1:** Results of generalized linear model examining the effects of spatial environment, day, and their interaction on female remating rates and mean offspring production in *Drosophila melanogaster*

Variable	(a) Remating			(b) Mean offspring		
	LR χ^2^	*df*	*p*	LR χ^2^	*df*	*p*
Environment	12.83	1	3.41 × 10^−4^	107.38	1	<1 × 10^−10^
Day	95.7	3	2.2 × 10^–16^	1217.96	3	<1 × 10^−10^
Day × environment	17.12	3	6.67 × 10^−4^	88.97	3	<1 × 10^−10^

**Table 2:** Results of Mann–Whitney and Cliff's delta (△) effect size statistics comparing the difference in median remating rates in female *Drosophila melanogaster* housed in simple or in complex spatial environments for each of the 4 days of the assay

Assay duration	Median		Mann–Whitney		Cliff's △	95% CI
	Simple	Complex	*W*	*p*		
24 hr	0.90	0.65	736.5	1.85 × 10^−05^	0.64	0.36 to 0.81
48 hr	0.90	0.90	358	0.16	−0.2	−0.46 to 0.08
72 hr	1.00	0.95	540	0.13	0.2	−0.07 to 0.44
96 hr	1.00	1.00	494	0.45	0.10	−0.16 to 0.34

**Table 3:** Results of Mann–Whitney and Cliff's delta (△) effect size statistics comparing the difference in median offspring production in female *Drosophila melanogaster* housed in simple or in complex spatial environments for each of the 4 days of the assay

Assay duration	Median		Mann–Whitney		Cliff's △	95% CI
	Simple	Complex	*W*	*p*		
24 hr	6.01	5.40	627.5	8.84 × 10^−3^	0.39	0.09 to 0.63
48 hr	6.00	7.80	189.5	1.23 × 10^−4^	−0.58	−0.78 to −0.58
72 hr	11.84	16.47	28.5	4.82 × 10^−10^	−0.93	−0.99 to −0.26
96 hr	12.88	17.27	101.5	2.67 × 10^−7^	−0.77	−0.92 to −0.46

**Figure 3:**
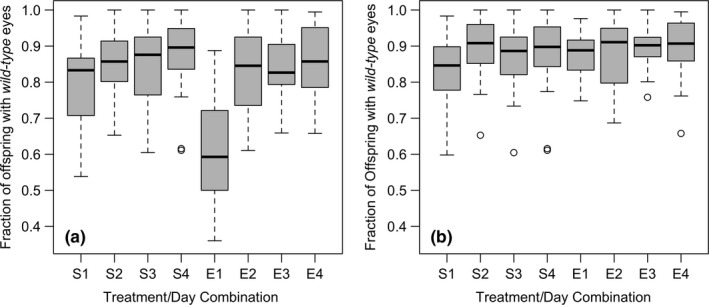
Boxplots illustrating the fraction of wild‐type to brown‐eyed offspring of *Drosophila melanogaster* in simple (S) or environmentally complex (C) chambers on each measured on each of the 4 days of the assay for (a) all females or (b) only those females that were classified as having remated. Boxplot components are as described in Figure 1

When examining all offspring produced by females, there was a significant effect of both environment and day on the fraction of wild‐type offspring in a female's brood, with the proportion of offspring sired by the initial (brown‐eyed) males decreasing over time, and a greater proportion of wild‐type offspring found in females housed in the simple environment. (Figure 3a, Table 4a). However, when the analysis was restricted to only remated females, those effects were no longer statistically significant (Figure 3b, Table 4b).

**Table 4:** ANOVA table for Scheirer–Ray–Hare analysis of fraction of wild‐type offspring in progeny of a) all female *Drosophila melanogaster* or b) only those females that remated that were housed in either a simple or in complex spatial environments for each of the 4 days of the assay

Factor	(a) All Females				(b) Only Remated Females			
	SS	*df*	*H*	*p*	SS	*df*	*H*	*p*
Environment	44,967	1	9.33	0.002	4,421	1	0.92	0.338
Day	213,585	3	44.31	1.293 × 10^−9^	40,253	3	8.35	0.039
Day x Environment	34,865	3	7.231	0.065	11,308	3	2.35	0.504
Residual	858,498	232			1,095,812	232		

### 3.2 Offspring fitness

The daughters sired by wild‐type males in simple and complex spatial chambers produced different numbers of offspring across the four days of the assay, revealed as a significant environment‐by‐day interaction (Table 5). Overall, daughters sired on later days were less fecund, especially those sired on the 4th day in the simple environment (Figure 2a), but there was no consistent differences when comparing offspring production between treatments on a day‐by‐day basis (Table 6). We found no significant effect of spatial environment or day on either the absolute or relative fitness of the sons sired by wild‐type males either in terms of how many females they successfully mated with, or the total offspring sired (Tables 7 and 8, Figure 2b).

**Table 5:** Results of GLMs examining the effects of spatial environmental complexity, day, and their interaction on the number of offspring produced by *Drosophila melanogaster* daughters whose parents were housed in either a simple or in a complex spatial environment for each of the 4 days of the assay

Variable	LR χ^2^	*df*	*p*
Environment	0.03	1	0.86
Day	17.43	3	5.77 × 10^−4^
Day x Environment	11.78	3	8.17 × 10^−3^

**Table 6:** Results of Mann–Whitney and Cliff's delta (△) effect size statistics comparing the difference in median offspring production in daughter *Drosophila melanogaster* from a parent generation that had been housed in either a simple or in a complex spatial environments for each of the 4 days of the assay

Assay duration	Median		Mann–Whitney		Cliff's △	95% CI
	Simple	Complex	*W*	*p*		
24 hr	33.6	32.8	491	0.55	0.09	−0.20 to 0.37
48 hr	32.8	34.7	319	0.05	−0.29	−0.53 to −0.01
72 hr	33.6	31.4	613	0.02	0.36	0.08 to 0.59
96 hr	27.0	30.7	334.5	0.09	−0.26	−0.52 to 0.05

**Table 7:** Results of statistical tests examining the effects of spatial environmental complexity, day, and their interaction on two measures of (absolute and relative) fitness (fraction of females in a group mated, and total offspring sired) of *Drosophila melanogaster* sons whose parents were housed in either a simple or in a complex spatial environments for each of the 4 days of the assay. Analyses on absolute data (a) were conducted using GLMs, while those on relative data (b) used the Scheirer–Ray–Hare method

(a)	Fraction of females mated to son				Total offspring sired by son			
Variable	LR χ^2^	*df*	*p*		LR χ^2^	*df*	*p*	
Environment	1.10	1	0.29		0.15	1	0.70	
Day	2.08	3	0.55		2.17	3	0.54	
Day x Environment	5.60	3	0.13		4.12	3	0.25	

(b)	Fraction of females mated to son				Total offspring sired by son			
Variable	SS	*df*	*H*	*p*	SS	*df*	*H*	*p*
Environment	0.005	1	0.14	0.71	3,768	1	0.79	0.37
Day	0.079	3	2.19	0.53	16,099	3	3.39	0.33
Day x Environment	0.146	3	4.05	0.26	26,632	3	5.61	0.13
Residual	8.379	232			1,087,569	232		

**Table 8:** Results of Mann–Whitney and Cliff's delta (△) effect size statistics comparing the difference in *Drosophila melanogaster* son fitness (measured as the fraction of females mated and in total number offspring sired) whose parents were housed in simple or in complex spatial environments for each of the 4 days of the assay

	Mann–Whitney			
Assay duration	*W*	*p*	Cliff's △	95% CI
(a) Fraction of females in vial that mated with son				
24 hr	336.5	0.09	−0.25	−0.51 to 0.04
48 hr	404.5	0.50	−0.10	−0.38 to 0.20
72 hr	544	0.16	0.21	−0.09 to 0.47
96 hr	407.5	0.53	−0.09	−0.34 to 0.17

(b) Total offspring sired by son				
24 hr	328.5	0.07	−0.27	−0.52 to 0.03
48 hr	448.5	0.84	−3.3 × 10^−3^	−0.28 to 0.28
72 hr	534	0.22	0.19	−0.11 to 0.46
96 hr	494.5	0.52	0.10	−0.19 to 0.37

## 4 DISCUSSION

Environmental spatial complexity has the potential to shape a population's evolutionary trajectory by changing the opportunity for sexual selection and/or conflict to be manifested. Here, we show that in *D. melanogaster*—an important model species for the study of sexual conflict and sexual selection—that an increase in environmental spatial complexity is associated with substantially increased offspring production in females, but had apparently no significant indirect genetic benefits that could be detected in the next generation. These patterns are consistent with the prediction that increased spatial complexity may free females from some of male‐induced harm that they experience (Yun et al., 2017). Our results help provide a better understanding of the factors that shape sexual selection's role as a potential agent of adaptive evolution and provides additional context for recent studies in this field.

In our first assay, we compared female performance in complex and simple environments by measuring cumulative remating rates and offspring production. Remating rates differed between the treatments, with the largest differences observed on the first day of the assay and decreasing in magnitude thereafter. Fecundity was higher for females in the simple environment on the first day of the assay; however, on every subsequent day of the trial, female fecundity was significantly higher in females in complex environments. In *D. melanogaster*, female fecundity is strongly influenced by the number (Kuijper, Stewart, & Rice, 2006) and/or timing of matings (Long, Pischedda, Nichols, & Rice, 2010). Young females may exhibit a short‐term “boost” to their fecundity following remating as a result of the extra dosage of Accessory Gland Proteins (ACPs; Long et al., 2010), so in the simple environment (where there was more remating), females would have had extra short‐term stimulation to produce offspring. However, the subsequent, higher fecundity of females in the complex environment is likely the direct and/or indirect result of decreased male‐induced harm experienced (Kuijper et al., 2006; Partridge & Fowler, 1990; Pitnick & García‐González, 2002). If it is harder in the complex environments for males to pursue and harass females, females will incur less damage from unwanted courtship/copulations (Partridge & Fowler, 1990), avoid wasting energy evading these males (Long et al., 2009), and have more time to feed (as fecundity in *D. melanogaster* females often being limited by food availability [see Chapman & Partridge, 1996; Linder & Rice, 2005; Stewart, Morrow, & Rice, 2005]). In our typical laboratory environments (i.e., simple vials), male‐induced harm is often strongly biased toward females of high potential fitness (Long et al., 2009), so if increased spatial complexity interferes with harassment, then these females will be more likely to produce more offspring. The lower average remating rates in experimental females also imply these females may experience less harm through copulation (Chapman, Liddle, Kalb, Wolfner, & Partridge, 1995; Grieshop & Polak, 2014; Kamimura, 2007), and/or decreased exposure to the physiologically modifying (and toxic) ACPs, than those females in the simple environment (Mueller, Page, & Wolfner, 2007; Pischedda, Stewart, Little, & Rice, 2010; Ram, Ji, & Wolfner, 2005; Wigby & Chapman, 2005; Wolfner, 2002). Although an initial consideration of sperm competition outcomes (fraction of offspring in a vial that were wild‐type) suggested a significant impact of day and environment, when analysis was restricted to only those females that were deemed remated, we saw no significant effect of these factors. If changing spatial complexity altered female access to “high quality” males, then one might reasonably expect an increased bias in the number of offspring sired by such males to be represented in her progeny (Snook, 2005) either through cryptic female choice or through altered sperm competition dynamics. Similarly, if there were systematic differences in the traits of those males that were successful at remating in the two environments (which might conceivably favor different levels of investment to pre‐vs.‐postcopulatory traits, Parker & Pizzari, 2010), then these differences should presumably be seen in their proportional representation in the offspring in the next generation). The absence of such differences between treatments (once differences in remated rates are taken into account) is circumstantial evidence against the male‐driven model of selection.

When examining the fitness of the offspring produced via remating in both environments, we looked for evidence whether increased spatial complexity resulted in offspring who were of better reproductive competitive ability. In the case of daughters, we saw a significant interaction between treatment and assay day (Table 5) but the specific differences in fecundity were not in any consistent manner that would be suggestive of differences in indirect genetic benefits (Table 6, Figure 3a). Overall, we observed lower fecundity of daughters produced by mothers from the later days of the trial. By the end of the assay, females in both treatments are potentially in worse physiological condition than when they started, due to senescence and/or male‐induced harm. This might be manifested in the next generation's phenotype through a maternal effect of decreased resources allocation to offspring (Azevedo, French, & Partridge, 1997), by manipulation by their mates to change reproductive investment patterns (Pischedda et al., 2010). It is possible that such a change in maternal effect might obscure any (increasing) indirect genetic benefits associated with remating by females. However, it should be noted that even on the 1st day of the assay, when mother fecundities were most similar (Figure 1c), and the net harm experienced by females was presumably the smallest between treatments, that no differences in daughter fecundity were observed. Interestingly, Garcia‐Gonzalez and Dowling (2015) have reported that *D. melanogaster *females that engaged in more polyandry saw *greater* offspring productivity in their daughters, presumably via an indirect genetic effect associated with the activity of ACPs from nonsire mates. If a similar indirect genetic effect was present in our study, this would have provided an additional boost to the fitness of daughters in the simple environments, where remating rates were higher, which should have made it easier to detect differences between treatments. In the absence of a significant spatial environmental complexity on daughter fitness, we posit that any effects on the manifestation of indirect genetic benefits seen in daughters are small to negligible in magnitude (and are dwarfed by the size of the direct effects of the males on the fecundity of mothers). Among males, we also saw no significant effect of treatment, trial day, or their interaction on individual reproductive success. If increased complexity facilitated females mating with higher quality males, we would have predicted some difference in the offspring fitness (the sons in particular), with the same caveats discussed above regarding daughter fitness. Thus, overall, we saw little evidence that increased spatial complexity enhanced adaptive evolution via indirect effects.

Our results dovetail with recent studies that have examined how spatial ecology can influence sexual selection/conflict dynamics and its consequence for adaptive evolutionary change, (also using *D. melanogaster*). In the first study, Byrne et al. (2008) set out to measure whether the presence of a male‐free spatial “refuge” affected female‐remating rates and fecundity. Females (which had been raised on limited media and were consequently smaller than usual) were able to access the refuge area, while males (raised at low larval densities and were much larger than usual) could not. The presence of the refuge was associated with a ~25% decrease in remating rate, but no difference in female lifetime fecundity between treatments was detected. However, it is unclear to what extent these observations were influenced by the novel developmental environments used to obtain males and females or that females using the refuge could not access live yeast (an important resource associated with fecundity) and may have been trading‐off harassment and feeding. Furthermore, the relatively short duration of the assay (48 hr) may have obscured any differences that would have become apparent with more prolonged exposure, which is often the case with *D. melanogaster *(Kuijper et al., 2006; Partridge & Fowler, 1990). Next, MacLellan et al. (2009) tested the relative competitive performance of a single wild‐type male against single males from 10 different populations of *D. melanogaster* that each expressed a visible phenotypic marker in both a small and a large arena (that differed ~600× in volume). The reproductive success of the mutant males was greater in the smaller chamber than in the larger chamber in 9 of the 10 assays (3 significantly so). This was interpreted as reflecting the greater challenges posed to mutant males of searching for mates in the more spacious environment and suggests that males experienced stronger sexual selection due to the increased environmental complexity. However, in addition to the issue discussed above related to the short duration of this assay, it was not reported whether there were any differences in the fecundity of females in these two treatments, which potentially also differed in their ease of escaping persistently harassing males. A more recent study by Yun et al. (2017) set out to quantify the extent of male behavior, male–female interactions and female fecundity in groups of flies that housed in two vastly different environments (a “simple” standard *Drosophila* culture vial and a “complex” 1,650 ml cage outfitted with pipe cleaner structures and five dishes of food). Though a series of elegant and complimentary experiments, they found that the flies’ environment dramatically influenced how the sexes interacted, which had significant consequences on the phenotypic expression of female fitness. In *D. melanogaster*, males may attempt to selfishly maximize their own lifetime reproductive success (LRS) by biasing their courtship (and harm) toward larger, more intrinsically fecund females, ultimately depressing these females’ LRS (Chenoweth et al., 2015; Long et al., 2009). Yun et al. (2017) observed that in the small, simple vials, males were able to effectively bias their attention toward large‐bodied females, but in the larger, more complex, cages that large‐ and small‐bodied females received the same frequency of sexual interactions. Large females spent more time being harassed in the simple vials and were observed to spend less time feeding than when they were housed in the more complex cages, with the net result being that these females achieved greater realized fecundities in the cages compared to in the vials. Our investigation into female remating rates and fecundity are largely consistent with the results of Yun et al. (2017), as we saw evidence that a more spatially complex environment was associated with a lower initial remating rates, and greater offspring production as the days passed than in the simple vials.

The same two types of environmental chambers employed by Yun et al.’s (2017) assays were also used in an experimental evolution study by Singh, Agrawal, and Rundle (2017) to see whether deleterious mutations could be more efficiently purged in more spatially complex environments. In this project, the alleles in question comprised of 22 different gene‐disruption mutations, whose frequencies were tracked over 8–10 generations of culture. In 18 of the 22 mutation lines surveyed, that allele frequencies dropped more rapidly in the complex chambers, which is suggestive of stronger selection against those carrying the deleterious mutations (possibly though more effective mate choice by females). However, as with Yun et al. (2017), it is not possible to rule out the (in our opinion unlikely) chance that the changes associated with selection in the complex environment are not the result of the differences in the chamber microhabitats independent of spatial complexity (i.e., humidity, food availability). This potential confound was avoided in the experimental evolution assay by Colpitts, Williscroft, Sekhon, and Rundle (2017), who used populations housed in two comparable arenas (a “simple” 1,650‐ml container and a single petri dish with 10 ml of media, or a “complex” environment of the same volume but containing five petri dishes of media and pipe cleaners to add physical complexity) to track the frequency of recessive deleterious alleles (with visible phenotypic effects) over time. Over the course of the assay (8–14 generations), the deleterious allele frequencies decreased more slowly in the simple environment for 2 of the 4 mutant lines tested, which is (partially) consistent with the prediction that selection against deleterious effects is more efficient in the complex environment.

The results of our research are also potentially useful for understanding the mechanisms that led to the changes in the frequencies of deleterious mutations seen in Singh et al. (2017) and Colpitts et al.’s (2017) studies (and perhaps explain why in some circumstances frequencies of these alleles did not change). Our assay strongly suggests that (at least in *D. melanogaster*) selection changes in spatially complex environments through a reduction in the “gender load” (sensu Rice & Chippindale, 2002) resulting from reduced male‐induced harm, rather than via enhanced sexual selection acting directly on males. This is born out of our observation that in more complex environments, females remated less and had greater offspring production (which is consistent with the results of Yun et al., 2017), and there was no increase in the reproductive success of either sons or daughters produced. Thus, the more efficient purging of deleterious mutations in the spatially complex treatments of Singh et al. (2017) and Colpitts et al.’s (2017) studies is likely to be due to females of greater reproductive potential being able to realize greater fecundities under reduced male harassment (Long et al., 2009; Yun et al., 2017). However, if that is the case, what might explain the *lack* of differences in allele frequencies in the complex and simple environment for some of the mutations they assayed? We hypothesize that this may be (in part) due to the specific fitness‐associated effects of the mutations used, and the design of their experimental evolution protocol, in which flies were not moved into the experimental chambers until the 11th or 12th day of each generation. Since females begin to eclose as adults starting ~9 days after being laid (and males starting ~the 10th day), adult flies in their studies potentially spent between 1–3 days interacting (and mating) in their initial culture chamber (a standard, simple vial environment), which our study showed can have significant negative effects on female fitness. However, the intensity of interactions during that phase might be lessened if the deleterious mutation present resulted in slower developmental rates and/or males with inferior courting abilities, resulting in fewer and/or less persistent males during those early days of the adult phase in each generation. As a consequence, females could experience relatively less (cumulative) harm before entering either the “experimental” simple or complex environments, where the “relief” of more spatial complexity can have its effect. However, if the mutations have no meaningful impact on male development or courtship, then females may enter the experimental phase of the selection protocol already too physiologically damaged from their early adult experiences for any benefits associated with increased spatial complexity to have a measurable effect on their fitness. It is worth considering that in Colpitts et al.’s (2017) study, the two mutations that were more effectively purged in the complex environment were *white* (which results in severely impaired vision) and *plexus* (which changes wing morphology) while those trials that showed no difference were *brown *and *sepia* (where the effect of the eye phenotype produced may not be as deleterious). Future studies should focus on examining how the relationship between specific mutations, their phenotypic effects on male development and courtship rates, and how this may affect their interactions with females of different potential fecundities. Such studies would help in understanding the situations under which sexual selection acts synergistically with natural selection to purge deleterious alleles from the gene pool.

Our current study has its own set of limitations which are worth considering. First, fruit flies (especially laboratory‐reared populations) are well known for their expression of intersexual conflict (Arnqvist & Rowe, 2005; Byrne et al., 2008; Chapman et al., 1995) so the differences we observed may not necessarily be seen in other species, and/or other populations that are raised under different conditions. Secondly, the exact nature and magnitude of the change(s) in selective pressures associated with differences in spatial complexity will likely depend on the specific characteristics of the structural elements in the environment. Culturing our laboratory populations in vials has undoubtedly resulted in them experiencing a dramatically simpler environment than they might encounter in the wild, or even in a population cage environment. Our method of introducing spatial complexity by adding a transparent folded acetate insert produced an effect, but is not clear how much/little environmental modification is needed to have an effect on mating dynamics, and is a promising avenue for future studies. Thirdly, in our assay we did not measure egg‐to‐adult survivorship of the offspring, and thus, we could be missing a potentially important source of indirect benefits. However, flies in our laboratory population, reared at standard densities, typically have a very high survivorship (Long, Montgomerie, & Chippindale, 2005), so it is unlikely that this is a major factor. While our four‐day assay length was designed to replicate the timeframe of adult male and female encounters that is typical in our laboratory setting (Martin & Long, 2015), a longer experimental duration might have provided more insight to both direct and indirect effects of spatial complexity on increasingly older flies. Furthermore, while our estimates of offspring reproductive competitive success were designed to replicate (as feasibly as possible) the competitive conditions that present in the normal culture environment IV population, it is possible that a different assay design might have yielded different results. Specifically, our decision to measure all son fitnesses in simple vials could be an issue if the (pre and/or post) male reproductive traits favored by selection in the complex environment are different from those favored in the simple one. However, in such a case one might have predicted to see an overall *worse* performance of those sons sired in the complex environment compared to those who had been sired in the simple environment, while we ultimately observed no significant differences between these groups.

Environmental variation is ubiquitous in natural systems, so most species have presumably evolved under heterogeneous selective pressures (Łukasik et al., 2006). Here, we show how increasing the spatial complexity of the environment in which selection takes place resulted in decreased remating rates and a corresponding increase in the average female reproductive output. This relationship is likely mediated by changes in male and female encounter rates and the availability of refuge sites for females experiencing costly harassment from persistent males. If environmental complexity was correlated with the strength of sexual selection, then successful males in the experimental treatment should have sired better quality offspring than those with less selection pressure; however, parental environmental complexity was not associated with differences in the fitness of their offspring. The net effect of sexual selection on the adaptive evolution is a long‐standing source of debate among evolutionary biologists (Berger et al., 2016; Candolin & Heuschele, 2008). Using an experimental evolution approach, Chenoweth et al. (2015) examined changes in allele frequencies in populations where the opportunity for natural and sexual selection was both manipulated. Surprisingly, while they saw similar, complementary, changes in certain allele frequencies when sexuals and natural selection acted independently, this was not the case when both processes operated simultaneously in a population. Follow‐up behavioral assays suggested that targeted male harassment of high quality females, arising from sexual conflict, hindered or even thwarted the species’ adaptive evolution. Thus, if increases in spatial complexity in an environment reduce harassment (as in Yun et al., 2017), the weakening of the “cost of attractiveness” may lead to the synergy between natural and sexual selection and promote adaptive evolution. A prolonged period of existence in a spatially complex/low‐harm environment may permit sexual selection to act more efficiently against those individuals carrying deleterious mutations (as in MacLellan et al., 2009). Overall, our research contributes to a better understanding how sexual selection operates and highlights the importance of considering environmental context both in laboratory‐reared and in wild populations when measuring the effects of sexual selection.

## CONFLICT OF INTEREST

The authors declare no conflict of interest.

## ETHICAL APPROVAL

This study did not require approval from an ethics committee.

## AUTHOR CONTRIBUTIONS

TAFL and HLM jointly conceived the experiments, collected the data, performed the analyses, and wrote the manuscript.

## Data Availability

Data have been deposited to Dryad: https://doi.org/10.5061/dryad.5mv5kt5.
