# Corrigendum

**DOI:** 10.1002/ece3.6559

**Published:** 2020-08-10

In Malek and Long (2019), owing to an error with the handling of the data files by Dryad, the DOI originally provided in the Data Accessibility statement is no longer active. Readers can now access the data using the newly assigned DOI as printed below:

https://doi.org/10.5061/dryad.00000001t
